# The treatment based on calcineurin inhibitors and their conversion to sirolimus alter the morphology in the rat testis

**DOI:** 10.1038/s41598-025-26892-3

**Published:** 2025-11-28

**Authors:** Marta Grabowska, Katarzyna Michałek, Karolina Kędzierska-Kapuza, Kamil Gill, Małgorzata Piasecka

**Affiliations:** 1 Department of Histology and Developmental Biology, Faculty of Health Sciences, Pomeranian Medical University, Żołnierska 48, 71-210 Szczecin, Poland; 2 Department of Physiology, Cytobiology and Proteomics, West Pomeranian University of Technology in Szczecin, Szczecin, Poland; 3 Department of Internal Medicine, Endocrinology and Diabetology, National Medical Institute of the Ministry of Interior Affairs and Administration , Wołoska 137, 02-507 Warsaw, Poland

**Keywords:** Testis, Rat, Sirolimus, Calcineurin inhibitors, Apoptosis, Proliferation, Cell biology, Diseases, Medical research

## Abstract

The effects of three-drug immunosuppressive regimens on the testis are still not fully known. The aim of this study was to evaluate the long-term effects of immunosuppressive protocols based on calcineurin inhibitors (CNIs) and their conversion to monotherapy with sirolimus on morphology, proliferation, and nuclear DNA fragmentation in the male gonad using an experimental model. For 6 months, male Wistar rats were treated with cyclosporin A (CsA), tacrolimus (FK-506), sirolimus (SRL), mycophenolate mofetil (MMF), and prednisone (Pred). The following protocols were used: CMP (CsA, MMF, and Pred), CMP/S (CsA, MMF, and Pred with conversion to SRL), TMP (FK-506, MMF, and Pred), and TMP/S (FK-506, MMF, and Pred with conversion to SRL). Morphological analyses, including morphometric analysis, immunohistochemical evaluation for proliferation (Ki67), and nuclear DNA fragmentation using the terminal deoxynucleotidyl transferase dUTP nick-end labelling (TUNEL) method in the testis, were conducted. In the testes of all the experimental groups, disorders in the organization of the seminiferous epithelium, a lower diameter and area of seminiferous tubules, and a lower percentage of Ki67-positive germinal cells than those in the control group were observed. A lower height of the seminiferous epithelium in the CMP, CMP/S and TMP/S groups than in the control group was noted. A higher percentage of TUNEL-positive germinal cells was detected in the CMP and TMP groups. In the groups with conversion to SRL, a lower diameter and area of seminiferous tubules and height of the seminiferous epithelium (TMP/S vs. TMP) and a lower percentage of Ki67- and TUNEL-positive cells (TMP/S vs. TMP; CMP/S vs. CMP) were revealed. The long-term administration of CNIs in multiple regimens can influence the course of spermatogenesis in the testes of rats, which manifests as morphological alterations in the seminiferous epithelium, vascular accumulation of collagen fibres in interstitial tissue, decreased proliferation of germinal cells and increased nuclear DNA fragmentation related to the apoptotic process. The conversion of treatment with CNIs to SRL also had adverse effects on the seminiferous epithelium; however, it had positive antiapoptotic effects. The obtained results may allow for a better understanding of changes in men undergoing immunosuppressive therapy and a preliminary assessment of which drug combination would be most beneficial in specific clinical cases.

## Introduction

Immunosuppressive drugs are a class of medications that are responsible for inhibiting or reducing the intensity of the immune response^1^. They are used in therapy for recipients of vascularized organ transplants, as well as in the treatment of autoimmune and inflammatory diseases of various aetiologies. In the case of transplantation, the main goal of immunosuppressive therapy is to prevent graft rejection by modulating the host immune response, which significantly improves patient survival^2,3^.

In clinical practice, multidrug protocols are used for organ transplant patients, which include calcineurin inhibitors (CNIs), such as cyclosporine A (CsA) and tacrolimus (FK-506); purine synthesis inhibitors, such as mycophenolate mofetil (MMF); mammalian target of rapamycin (mTOR) kinase inhibitors, such as sirolimus (SRL), also called rapamycin; and glucocorticoids. The administration of multidrug protocols has contributed to significant improvements in the condition of transplant patients^4,5^.

The introduction of CNIs into clinical practice in the 1980s significantly increased patient survival and reduced episodes of acute rejection. Despite these benefits, prolonged use of CNIs is associated with serious side effects, including nephrotoxicity^6^, hepatotoxicity^7^, posttransplant diabetes mellitus, and malignancy incidence^8^. In the case of significant renal impairment or other dysfunctions associated with CNI administration, a therapeutic strategy is proposed, including complete discontinuation of CNIs within a specified period after transplantation and the introduction of an alternative immunosuppressive drug or minimization of the maintenance dose of CsA. SRL can act as an effective substitute for CNI therapy in the later period after organ transplantation^9^. mTOR inhibitors have potent antiproliferative and immunomodulatory effects and a favourable safety profile with respect to nephrotoxicity. The use of SRL may also be associated with a reduced risk of developing malignancies in transplant recipients, which is particularly important in high-risk oncological patients^10^. Therefore, conversion from CNI therapy to SRL monotherapy may be a reasonable therapeutic alternative in many clinical situations^11^.

The long-term use of immunosuppressive drugs is associated with several adverse effects on various organs and systems, including an increased risk of cardiovascular diseases and malignancies^8,12,13^. Studies conducted on experimental models have also shown that these drugs may cause adverse changes in the male reproductive system that may lead to impaired spermatogenesis^14–16^. In humans, immunosuppressive drugs may cause disorders in male reproductive capacity in the form of changes in the secretion or action of hypothalamic‒pituitary‒gonadal axis hormones, spermatogenesis disorders, or reduced sperm motility and concentration^17–20^.

However, despite numerous experimental studies on the effects of single immunosuppressive drugs on the male reproductive system, only a few scientific reports have described the effects of using multidrug immunosuppressive regimens on the male gonad^21^. Notably, the long-term effects of CNI use in triple therapy or the consequences of conversion from CNI treatment to SRL monotherapy in the context of male gonad function have not been studied thus far, although the current therapeutic standard in transplant recipients is the use of multidrug regimens. Therefore, the aim of this study was to evaluate the long-term effects of multidrug immunosuppressive protocols based on CNIs and their conversion to monotherapy with SRL on morphology, cell proliferation, and nuclear DNA fragmentation in the rat male gonad.

## Results

### Morphological evaluation

**Fig. 1:**
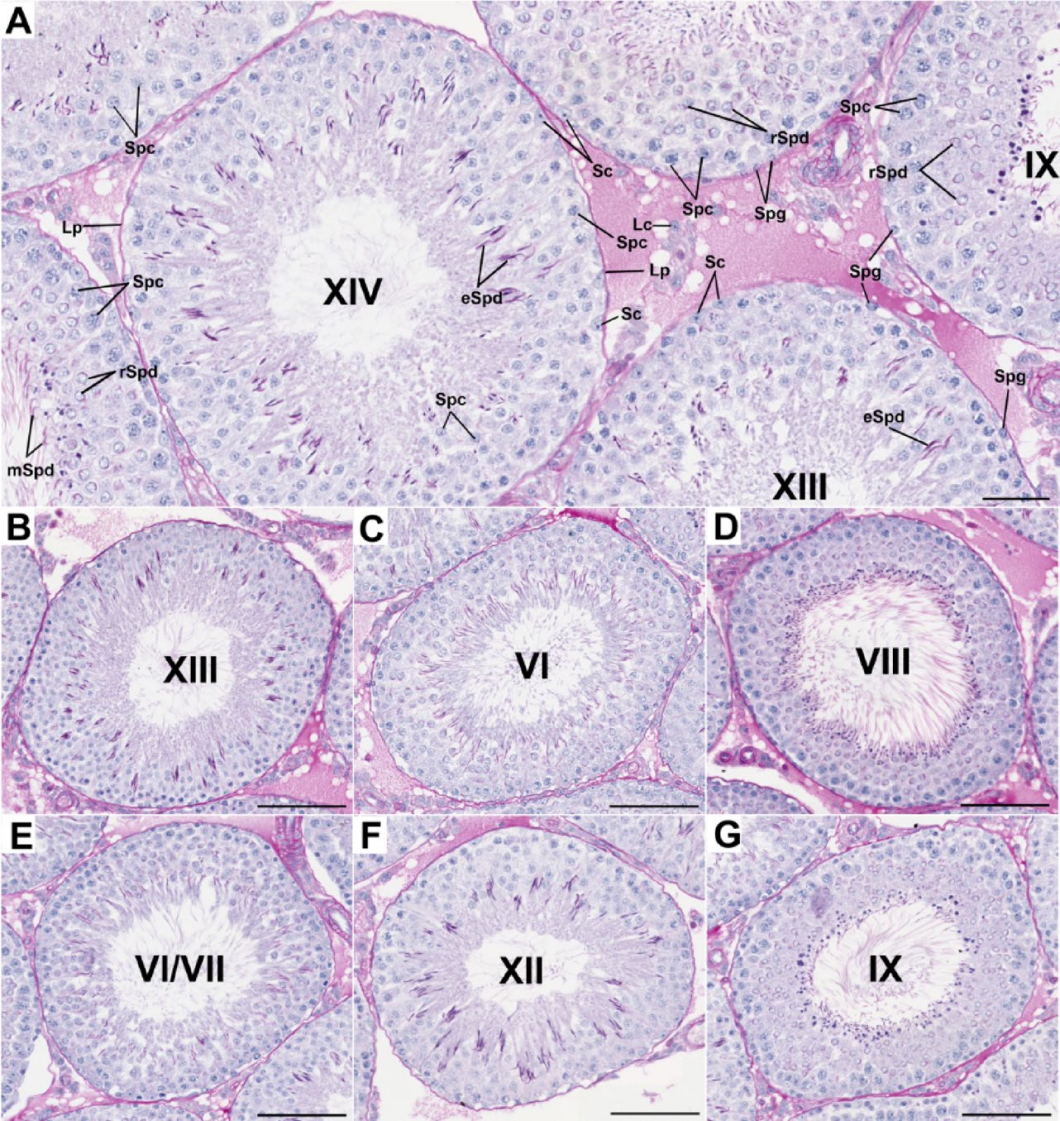
Representative light micrographs (PAS staining) presenting cross-sections through the seminiferous tubules of testes in the control group (**A**–**G**). The seminiferous tubules with normal organization of the germinal epithelium; different stages of the seminiferous epithelium cycle (VI, VII, VIII, IX, XII, XIII, XIV); spermatogonia (Spg), spermatocytes (Spc), rounded—early spermatids (rSpd) and elongating—late spermatids (eSpd), releasing mature elongated spermatids (mSpd), the nucleus of Sertoli cells (Sc), and Leydig cells (Lc) in the interstitial tissue; thin lamina propria (Lp) of the seminiferous tubules.

**Fig. 2:**
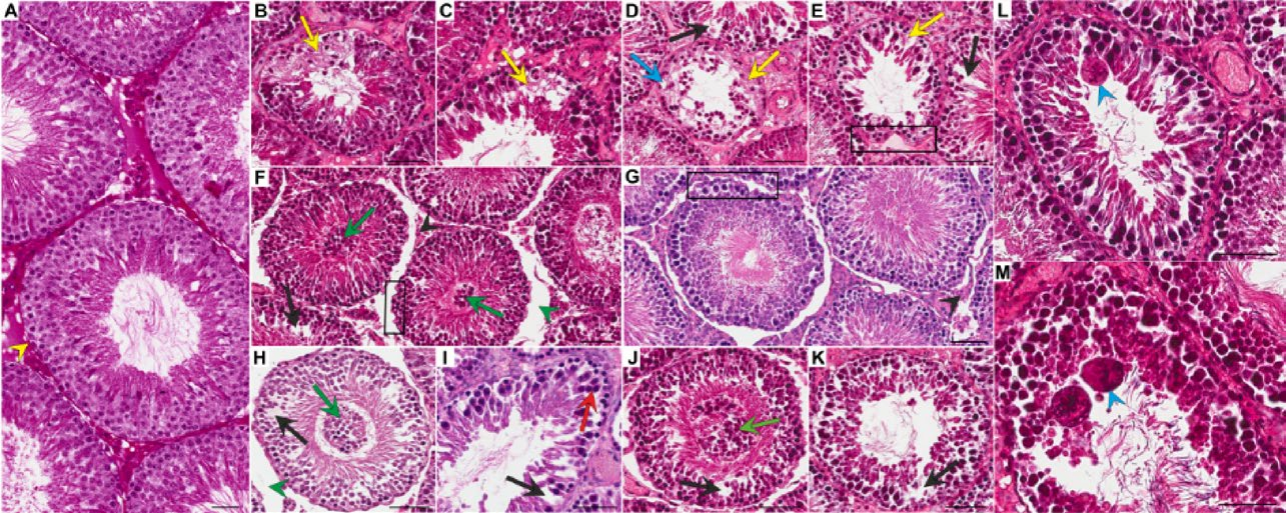
Effect of three-drug immunosuppressive regimens based on calcineurin inhibitors and conversion to sirolimus monotherapy on the morphology of the rat testis. Representative light micrographs (H&E staining) showing cross-sections through the seminiferous tubules of testes in the control group (**A**) and experimental groups: CMP (**B**, **C**, **L**), CMP/S (**D**, **E**, **F**), TMP (**G**, **H**, **I**) and TMP/S (**J**, **K**, **M**). No significant morphological changes or seminiferous tubules with normal thin lamina propria (**A**, yellow arrowhead) were observed in the control group. Disorders in the organization of the seminiferous epithelium (**B**–**M**), including inappriopriate arrangement of germ cells in the seminiferous epithelium (**B**–**E**, yellow arrows), the presence of prematurely sloughed cells of the spermatogenic pathway into the lumen (**F**, **H**, **J**, green arrows), a reduced number of germ cells and numerous empty spaces in the epithelium (**D**–**F**, **H**–**K**, black arrows), intercellular vacuolization (**D**, blue arrow), small multinuclear cells (**I**, red arrow), giant multinuclear cells (**L**, **M**, blue arrowheads), irregular and folded walls of tubules (**E**–**G**, black frames), shrinkage of interstitial tissue (**F**, **G**, black arrowheads) and loss of connections between the wall of tubules and interstitial tissue, resulting in empty spaces in the interstitial area (**F**, **H**, green arrowheads) in the experimental groups. Scale bar—50 µm. CMP, rats treated with cyclosporin A, mycophenolate mofetil, and prednisone for 6 months; CMP/S, rats treated with cyclosporin A, mycophenolate mofetil, and prednisone for the first 3 months of the experiment and sirolimus for the last 3 months; H&E, haematoxylin and eosin; TMP, rats treated with tacrolimus, mycophenolate mofetil, and prednisone for 6 months; TMP/S, rats treated with tacrolimus, mycophenolate mofetil, and prednisone for the first 3 months of the experiment and sirolimus for the last 3 months.

Morphological analysis of rat testes (based on the PAS and H&E staining) in the control group revealed that the seminiferous tubules had thin lamina propria and were located close to each other. All generations and layers of germ cells, which are typical for the proper stages of the seminiferous epithelium cycle, were present. Spermatogonia, spermatocytes, early rounded spermatids, and elongating as well as late elongated spermatids and Sertoli cells were observed. Furthermore, testicular spermatozoa—mature elongated spermatids released into the lumen of tubules—were found. Moreover, the interstitial spaces between convoluted tubules were small and contained clusters of Leydig cells with vesicular nuclei, blood vessels, and loosely formed connective tissue (Figs. 1A–G, 2A).

Long-term immunosuppressive treatment using three-drug protocols involving calcineurin inhibitors (CMP and TMP groups) and conversion to SRL monotherapy (CMP/S and TMP/S groups) resulted in visible changes in the morphology of the seminiferous epithelium in experimental animals. A normal thin lamina propria was found (Fig. 2B–M). In all the experimental groups (CMP, CMP/S, TMP, and TMP/S), disorders in the organization of the seminiferous epithelium, including inappropriate arrangement of germ cells in the seminiferous epithelium, the presence of prematurely sloughed cells of the spermatogenic pathway into the lumen, a reduced number of germ cells, numerous empty spaces in the epithelium, intercellular vacuolization, and small and giant multinuclear cells in the seminiferous epithelium, were observed. Moreover, the walls of some tubules were irregular and folded. Shrinkage of interstitial tissue and loss of connections between the walls of tubules and interstitial tissue resulted in empty spaces in the interstitial area (Fig. 2B–M).

Furthermore, in the animals treated with CNI-based regimens in which these protocols were converted to SRL (CMP/S and TMP/S groups), detachment of germ cells into the lumen of seminiferous tubules was observed more often (Fig. 2F,J). Moreover, residual bodies at the adluminal surface were noted (Table 1).

A significantly higher percentage of tubules with markedly reduced number of germ cells was found in all experimental groups of rats (CMP, CMP/S, TMP, and TMP/S) compared to the control group (*p* < 0.001, *p* = 0.006, and *p* = 0.010, respectively). In animals treated with CsA-based protocols (CMP and CMP/S) and FK506-based regimens converted to SRL (TMP/S) a significant increase in the percentages of tubules with inappropriate arrangement of germ cells (*p* < 0.001, *p* = 0.009, and *p* = 0.048, respectively), and multinuclear cells (*p* < 0.001, respectively) were revealed compared to the control group. In rats treated with CNI-based regimens converted to SRL a significant increase in the percentages of tubules with the presence of prematurely sloughed cells into the lumen (*p* = 0.004, and *p* = 0.002, respectively), and irregular and folded walls (*p* < 0.001, and *p* = 0.044, respectively) were noted compared to the control group. A significantly higher percentage of tubules with empty spaces in the epithelium was observed in animals treated with CsA-based protocols (CMP and CMP/S) compared to the control group (*p* < 0.001, and *p* = 0.021, respectively). In turn, a significantly higher percentage of tubules with intercellular vacuolization was found only in rats treated with FK506-based regimens converted to SRL (TMP/S; *p* = 0.037) (Table 1).

**Table 1:** Comparison of the percentage of seminiferous tubule with selected disorders in rats between the control and experimental groups (CMP, CMP/S, TMP, and TMP/S).

Parameter	C	CMP	CMP/S	TMP	TMP/S
	% tubules	% tubules	% tubules	% tubules	% tubules
Inappropriate arrangement of germ cells	0.23 ± 0.56	6.14^b^ ± 2.91	6.99^b^ ± 2.26	4.06 ± 2.88	8.69^a^ ± 3.34
The presence of prematurely sloughed cells into the lumen	0.48 ± 0.75	4.09 ± 1.49	11.66^b^ ± 11.05	6.08 ± 8.73	10.23^b^ ± 6.71
markedly reduced number of germ cells	0.00 ± 0.00	6.13^a^ ± 3.12	5.24^b^ ± 2.20	4.95^b^ ± 3.03	6.06^a^ ± 2.04
Empty spaces in the epithelium	6.49 ± 2.25	18.19^a^ ± 6.46	15.11^b^ ± 4.95	11.25 ± 2.42	10.65 ± 4.90
Intercellular vacuolization	1.94 ± 2.07	5.24 ± 2.24	4.60 ± 1.40	3.82 ± 1.34	5.49^b^ ± 2.61
Multinuclear cells	0.00 ± 0.00	6.84^a^ ± 2.63	7.71^a^ ± 2.01	3.10 ± 2.14	5.64^a^ ± 1.70
Irregular and folded walls	4.09 ± 1.46	12.44 ± 5.70	23.61^a,c^ ± 9.14	12.97 ± 3.39	14.74^b^ ± 7.70

^a^*p* < 0.001 versus control; ^b^*p* < 0.05 versus control; ^c^*p* < 0.05 versus CMP; CMP, rats treated with cyclosporin A, mycophenolate mofetil, and prednisone for 6 months; CMP/S, rats treated with cyclosporin A, mycophenolate mofetil, and prednisone for the first 3 months of the experiment and sirolimus for the last 3 months; TMP, rats treated with tacrolimus, mycophenolate mofetil, and prednisone for 6 months; TMP/S, rats treated with tacrolimus, mycophenolate mofetil, and prednisone for the first 3 months of the experiment and sirolimus for the last 3 months;

### Analysis of collagen

**Fig. 3:**
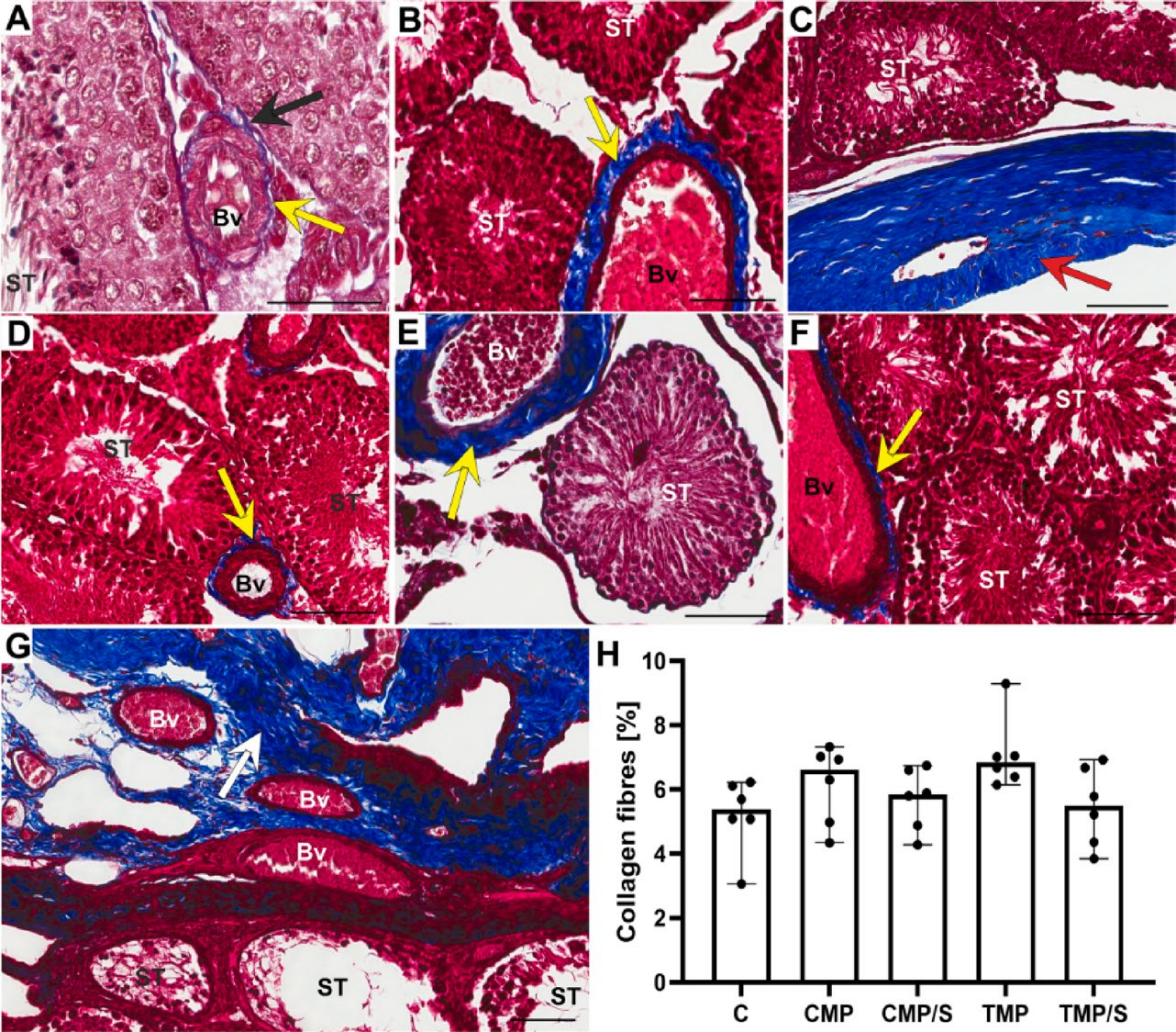
Effect of multidrug immunosuppressive protocols based on calcineurin inhibitors and conversion to sirolimus on the localization and percentage of collagen in the rat male gonad. Representative light micrographs (Masson’s trichrome staining) showing blue-stained collagen fibres in the control group (**A**) and experimental groups: CMP (**B**, **C**, **G**), CMP/S (**D**), TMP (**E**) and TMP/S (**F**). Collagen fibres in the interstitial area around the blood vessels (**A**, **B**, **D**‒**F**, yellow arrows) and in the lamina propria of the seminiferous tubules (**A**, black arrow). Abundant collagen fibres in the outer part of the tunica albuginea (**C**, red arrow) and the inner part called the tunica vasculosa (**G**, white arrow) covering the testis. Bv, blood vessels; ST, seminiferous tubules. Scale bar, 50 µm. Comparison of the percentage of collagen (**H**) between the control and experimental groups (CMP, CMP/S, TMP, and TMP/S). The results are shown as medians and ranges. C, control group without any medication; CMP, rats treated with cyclosporin A, mycophenolate mofetil, and prednisone for 6 months; CMP/S, rats treated with cyclosporin A, mycophenolate mofetil, and prednisone for the first 3 months of the experiment and sirolimus for the last 3 months; TMP, rats treated with tacrolimus, mycophenolate mofetil, and prednisone for 6 months; TMP/S, rats treated with tacrolimus, mycophenolate mofetil, and prednisone for the first 3 months of the experiment and sirolimus for the last 3 months.

In both the control and experimental groups, Masson’s trichrome staining revealed collagen fibres in the outer part of the tunica albuginea and the inner part (tunica vasculosa) covering the testes. Delicate staining of the fibres was also observed in the lamina propria of the seminiferous tubules and in the interstitial area of the gonad around the blood vessels (Fig. 3A–G). In the experimental groups, compared to those in the control group, greater accumulation of collagen around the blood vessels was detected, whereas greater deposition of collagen fibres in the lamina propria was not detected. In the case of all analysed groups, there were no statistically significant differences (Fig. 3H).

### Testis weight and morphometric analysis

A significantly lower testes weight (*p* = 0.016) was found in the CsA-based group that was converted to SRL (CMP/S) compared to the control group (Table 2).

**Table 2:** Comparison of testes weight, number and percentage of Sertoli cells (Sc), spermatogonia (Spg), spermatocytes (Spc) and spermatids (Spd) in cross-sectioned seminiferous tubules in rats between the control and experimental groups (CMP, CMP/S, TMP, and TMP/S).

Parameter	C	CMP	CMP/S	TMP	TMP/S
	Mean ± SD	Mean ± SD	Mean ± SD	Mean ± SD	Mean ± SD
Testes weight [g]	3.47 ± 0.40	3.17 ± 0.27	2.86^b^ ± 0.27	2.97 ± 0.35	2.95 ± 0.20
No. of Sc	12.87 ± 2.00	9.72^a^ ± 2.31	9.83^a^ ± 2.06	11.10^a^ ± 2.19	9.95^a^ ± 2.06
% of Sc	3.63 ± 0.55	3.40 ± 0.87	3.37 ± 0.61	3.35^b^ ± 0.80	3.44 ± 0.83
No. of Spg	24.08 ± 4.36	20.65^a^ ± 3.89	19.32^a^ ± 3.35	22.43 ± 4.90	19.18^a,c^ ± 3.19
% of Spg	6.81 ± 1.23	7.19 ± 1.36	6.69 ± 1.22	6.72 ± 1.44	6.58 ± 0.93
No. of Spc	108.48 ± 10.99	87.83^a^ ± 14.51	89.27^a^ ± 15.51	102.00 ± 14.41	90.25^a,d^ ± 17.35
% of Spc	30.62 ± 2.63	30.21 ± 2.63	30.37 ± 1.52	30.30 ± 1.79	30.47 ± 1.59
No. of Spd	208.67 ± 12.41	172.72^a^ ± 29.98	174.98^a^ ± 28.74	200.50 ± 24.70	175.23^a,c^ ± 27.37
% of Spd	58.94 ± 2.72	59.21 ± 2.97	59.58 ± 1.60	59.63 ± 2.13	59.51 ± 1.48

^a^*p* < 0.001 versus control; ^b^*p* < 0.05 versus control; ^c^*p* < 0.001 versus TMP; ^d^*p* < 0.05 versus TMP; C, control group without any medication; CMP, rats treated with cyclosporin A, mycophenolate mofetil, and prednisone for 6 months; CMP/S, rats treated with cyclosporin A, mycophenolate mofetil, and prednisone for the first 3 months of the experiment and sirolimus for the last 3 months; TMP, rats treated with tacrolimus, mycophenolate mofetil, and prednisone for 6 months; TMP/S, rats treated with tacrolimus, mycophenolate mofetil, and prednisone for the first 3 months of the experiment and sirolimus for the last 3 months; No. of Sc, number of Sertoli cells per tubule; No. of Spg, number of spermatogonia per tubule; No. of Spc, number of spermatocytes per tubule; No. of Spd, number of spermatids per tubule; % of Sc, percentage of Sertoli cells per tubule; % of Spg, percentage of spermatogonia per tubule; % of Spc, percentage of spermatocytes per tubule; % of Spd, percentage of spermatids per tubule.

**Fig. 4:**
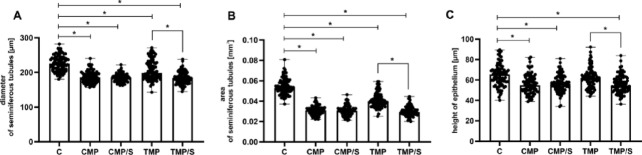
Effects of multidrug immunosuppressive protocols based on calcineurin inhibitors and conversion to sirolimus on the diameter (**A**) and area (**B**) of seminiferous tubules and the height (**C**) of the seminiferous tubule epithelium in male rat gonads. Comparison of the diameter and area of the seminiferous tubules and the height of the seminiferous tubule epithelium between the control and experimental groups (CMP, CMP/S, TMP, and TMP/S). The results are shown as medians and ranges. *p < 0.001 (Kruskal–Wallis test followed by Dunn’s multiple comparison post hoc test). C, control group without any medication; CMP, rats treated with cyclosporin A, mycophenolate mofetil, and prednisone for 6 months; CMP/S, rats treated with cyclosporin A, mycophenolate mofetil, and prednisone for the first 3 months of the experiment and sirolimus for the last 3 months; TMP, rats treated with tacrolimus, mycophenolate mofetil, and prednisone for 6 months; TMP/S, rats treated with tacrolimus, mycophenolate mofetil, and prednisone for the first 3 months of the experiment and sirolimus for the last 3 months.

Both in the case of the use of three-drug protocols based on calcineurin inhibitors and the conversion of treatment to SRL monotherapy, an unfavourable effect of these drugs on the morphometric parameters of the rat gonad was revealed. Analysis revealed a significantly lower diameter (Fig. 4A) and area of seminiferous tubules (Fig. 4B) in all experimental groups (CMP, CMP/S, TMP, and TMP/S) than in the control group (*p* < 0.001, respectively). Moreover, a significantly lower height of the seminiferous tubule epithelium (Fig. 4C) was noted in the CsA-based group with (CMP/S) and without conversion to SRL (CMP), and also in the FK506-based group with conversion to SRL (TMP/S) than in the control group (*p* < 0.001). A significantly lower diameter and area of seminiferous tubules and a lower height of the seminiferous tubule epithelium were also revealed in rats treated with FK506-based protocols converted to SRL than in animals treated with FK506-based protocols without conversion (TMP/S vs. TMP; *p* < 0.001, respectively). However, there were no significant differences in those parameters between the CsA-based groups (CMP/S and CMP).

A significantly lower number of Sertoli cells per tubule was revealed in all experimental groups of rats (CMP, CMP/S, TMP, and TMP/S) compared to the control group (*p* < 0.001, respectively). Additionally, animals treated with CsA-based protocols (CMP and CMP/S) and FK506-based regimens converted to SRL (TMP/S) showed a significant decrease in the numbers of spermatogonia, spermatocytes, and spermatids per tubule compared to the control group. Furthermore, a significantly lower number of spermatogonia, spermatocytes, and spermatids was observed in animals treated with FK506-based protocols converted to SRL compared to rats administered FK506-based protocols without conversion (TMP/S vs. TMP; *p* < 0.001 and *p* = 0.007, respectively). Regarding the percentage of the different cell types present in the seminiferous tubules, only rats treated with FK506-based protocols without conversion to SRL (TMP group) had a significantly lower percentage of Sertoli cells (*p* = 0.049) compared to the control group (Table 2).

### Immunostaining and the percentage of Ki67-positive cells

**Fig. 5:**
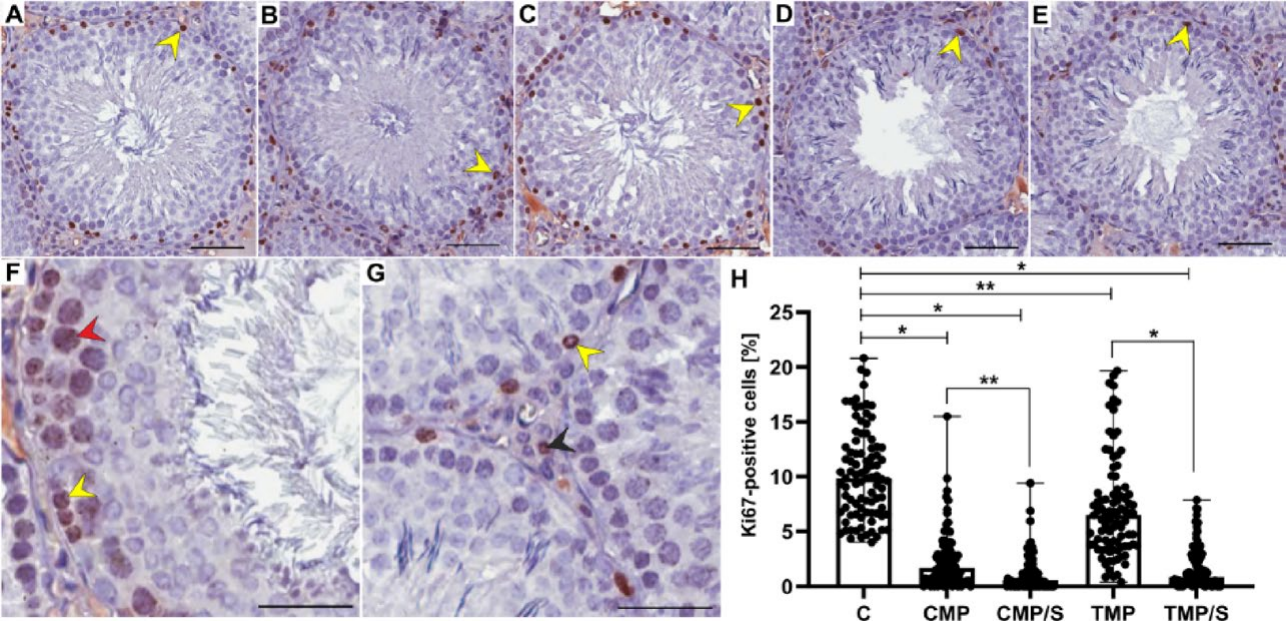
Effects of multidrug immunosuppressive protocols based on calcineurin inhibitors and conversion to sirolimus on the immunoexpression (**A**–**G**) and the percentage of Ki67-positive cells (**H**) in the seminiferous tubules of male rat gonads. Immunoexpression of Ki67 in spermatogonia (yellow arrowheads) and in primary spermatocytes (red arrowhead) in the control (**A**, **F**, **G**), CMP (**B**), CMP/S (**C**), TMP (**D**), and TMP/S (**E**) groups. Ki67-positive cells in the interstitial tissue (**G**). Scale bar, 50 µm. Comparison of the percentage of Ki67-positive cells (**H**) between the control and experimental groups (CMP, CMP/S, TMP, and TMP/S). The results are shown as medians and ranges. *p < 0.001; **p < 0.05 (Kruskal–Wallis test followed by Dunn’s multiple comparison post hoc test). C, control group without any medication; CMP, rats treated with cyclosporin A, mycophenolate mofetil, and prednisone for 6 months; CMP/S, rats treated with cyclosporin A, mycophenolate mofetil, and prednisone for the first 3 months of the experiment and sirolimus for the last 3 months; TMP, rats treated with tacrolimus, mycophenolate mofetil, and prednisone for 6 months; TMP/S, rats treated with tacrolimus, mycophenolate mofetil, and prednisone for the first 3 months of the experiment and sirolimus for the last 3 months.

In all examined seminiferous tubules in the control group and in the experimental groups of rats, immunostaining of Ki67 was observed in the form of brown-stained cell nuclei. Ki67 immunoexpression was detected mainly in spermatogonia; however, in some seminiferous tubules, Ki67 immunoexpression in primary spermatocytes was also noted (Fig. 5A‒G). Moreover, in the interstitial area, Ki67-positive Leydig cells and endothelial cells were also found (Fig. 5G).

In the seminiferous tubules of male rat gonads, the percentage of Ki67-positive cells was significantly lower in all experimental groups (CMP, CMP/S, TMP and TMP/S) than in the control group (*p* < 0.001, *p* = 0.017, respectively). Moreover, a significantly lower (*p* < 0.015 and *p* < 0.001, respectively) percentage of Ki67-positive cells was noted in animals treated with CNI-based protocols converted to SRL than in rats treated with non-converted protocols (CMP/S vs. CMP, and TMP/S vs. TMP) (Fig. 5H).

### Immunostaining and the percentage of TUNEL-positive cells

**Fig. 6:**
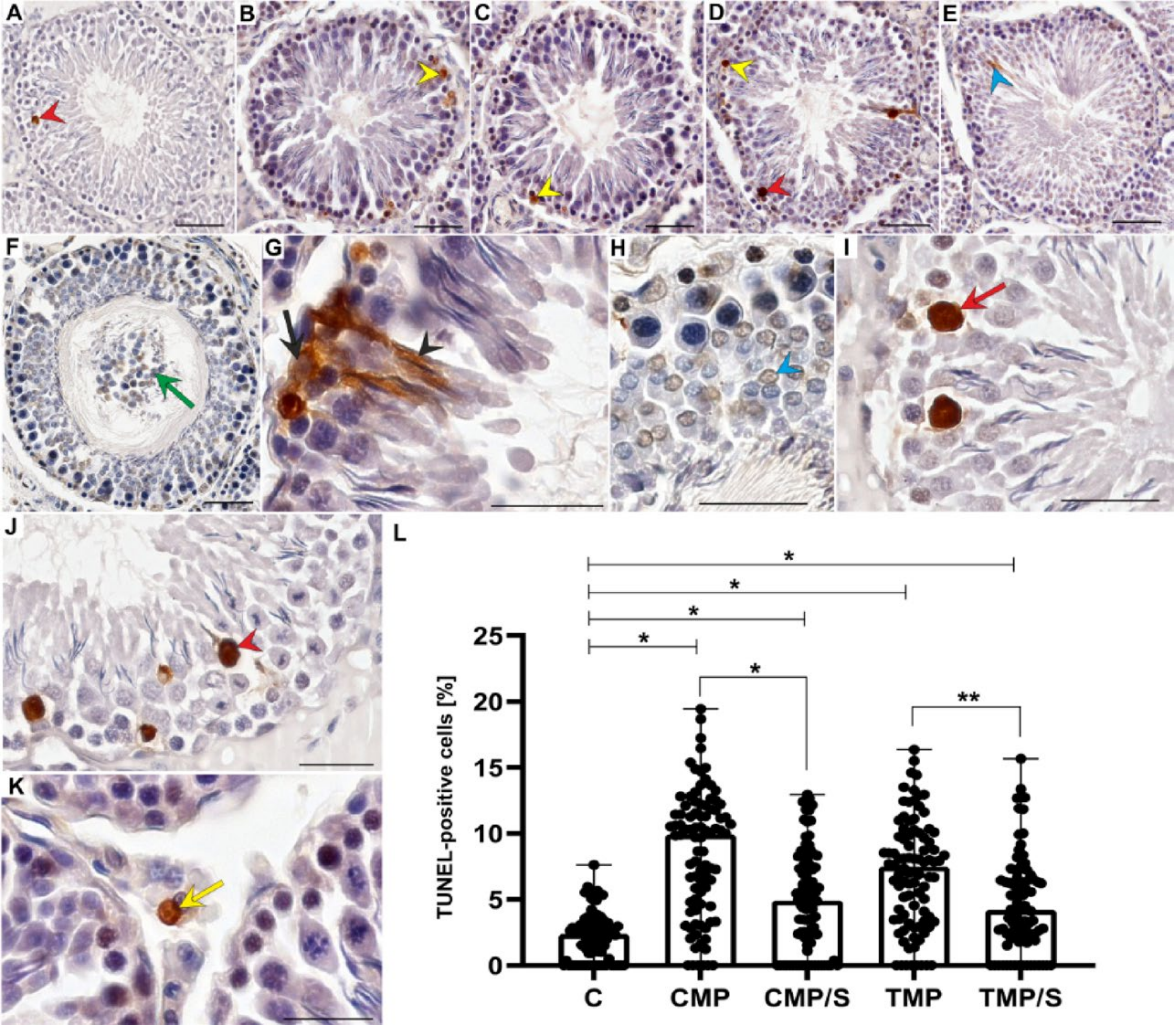
Effects of multidrug immunosuppressive protocols based on calcineurin inhibitors and conversion to sirolimus on the localization (**A**‒**K**) and percentage (**L**) of TUNEL-positive cells in the seminiferous tubules of male rat gonads. TUNEL-positive spermatogonia (yellow arrowheads), primary spermatocytes (red arrowhead), round (blue arrowhead) and elongated (black arrowhead) spermatids, giant multinuclear cells (red arrow), Sertoli cell (black arrow), and prematurely sloughed cells of the spermatogenic pathway into the lumen (green arrow) in the control (**A**, **J**, **K**) and CMP (**B**), CMP/S (**C**, **H**, **I**), TMP (**D**, **F**), and TMP/S (**E**, **G**) groups. TUNEL-positive Leydig cells were detected in the interstitial tissue (K, yellow arrow). Scale bar, 50 µm. Comparison of the percentage of TUNEL-positive cells (**L**) between the control and experimental groups (CMP, CMP/S, TMP, TMP/S). The results are shown as medians and ranges. *p < 0.001; **p < 0.05 (Kruskal–Wallis test followed by Dunn’s multiple comparison post hoc test). C, control group without any medication; CMP, rats treated with cyclosporin A, mycophenolate mofetil, and prednisone for 6 months; CMP/S, rats treated with cyclosporin A, mycophenolate mofetil, and prednisone for the first 3 months of the experiment and sirolimus for the last 3 months; TMP, rats treated with tacrolimus, mycophenolate mofetil, and prednisone for 6 months; TMP/S, rats treated with tacrolimus, mycophenolate mofetil, and prednisone for the first 3 months of the experiment and sirolimus for the last 3 months.

In all the animals, TUNEL-positive cells (with nuclear DNA fragmentation) were characterized by brown-stained nuclei. TUNEL-positive germ cells within the seminiferous epithelium, mainly spermatogonia, were observed in all the groups. Moreover, TUNEL labelling was also revealed in spermatocytes, round and elongated spermatids, Sertoli cells and detached germ cells in the lumen of seminiferous tubules. In some tubules, TUNEL-positive giant multinucleated cells were also detected (Fig. 6A‒K).

In the seminiferous tubules of male rat gonads, the percentage of TUNEL-positive cells was significantly higher in all experimental groups (CMP, CMP/S, TMP and TMP/S) than in the control group (*p* < 0.001, respectively). Moreover, a significantly lower (*p* < 0.001 and *p* < 0.003, respectively) percentage of TUNEL-positive cells was noted in animals treated with CNI-based protocols converted to SRL than in rats treated with protocols without conversion (CMP/S vs. CMP, and TMP/S vs. TMP) (Fig. 6L).

## Discussion

This study seems to be crucial for the identification of morphological changes, which may be associated with disorders of fertility in patients following vascularized organ transplantation. Notably, this is the first report that examines the long-term influence of three-drug immunosuppressive protocols based on CNIs and their conversion to SRL monotherapy on the male gonads of rats.

In this study, we investigated the effects of long-term immunosuppressive drug administration in multidrug protocols, which provides insight into the therapeutic regimens used in people after organ transplantation. An attempt was made to precisely determine the direct effects of multidrug immunosuppressive treatment regimens on male rat gonads. For this purpose, the experiment was conducted on animals not subjected to organ transplantation. This approach allows for the elimination of confounding factors, such as the clinical condition of the donor, the ischaemia/reperfusion phenomenon, drug interactions or immunological factors.

The experimental animals received the drugs for a period of 6 months, which, assuming that the average lifespan of a rat is 2–3 years, corresponds to approximately 15 years of human life. Notably, previous studies on the effects of immunosuppression on the male gonad in experimental models were characterized by a much shorter duration of exposure to drugs than this experiment did^14,21^.

Notably, the oral route of immunosuppressant administration, which is the physiological form, was used in the present model. In contrast to this methodology, in studies by other authors, subcutaneous or intraperitoneal drug administration or gastric intubation are often used^4,15,16^.

The doses of immunosuppressive drugs used in our study were carefully selected to achieve blood concentrations within the established therapeutic range^22^, avoiding the toxic levels reported in previous investigations. For example, in the study conducted by Rovira et al.^15^, SRL levels measured 12 h after intraperitoneal administration reached as high as 38 ng/ml, which is considerably elevated. In contrast, our measurements taken 4 h following the oral administration of SRL in rats revealed significantly lower blood concentrations of 6.4 ng/ml.

The drug doses used in other experiments were also higher than those used in our study. The authors treated animals with CsA at doses of 40 mg/kg^23^ and 25 mg/kg body weight^4^. In turn, FK-506 was administered at a dose of 1 mg/kg^14^ or 0.8 mg/kg body weight^4^. On the other hand, the animals were treated with SRL at a dose of 1 mg/kg^15^ and 2, 4, or 6 mg/kg body weight^16^. It is worth noting that in immunosuppressive therapy, higher doses of a drug are typically used in monotherapy than when the same drug is part of a multidrug regimen. This approach aims to achieve the desired therapeutic effect while minimizing individual drug toxicity and leveraging synergistic or additive effects of combined agents.

The findings of the present study demonstrated that prolonged administration of CNI-based immunosuppressive therapy, as well as its subsequent conversion to SRL monotherapy, induced marked morphological alterations in the histoarchitecture of the seminiferous epithelium in the gonads of male rats. Among the observed irregularities were, among others, the inappropriate arrangement of germ cells, the presence of multinucleated cells within the seminiferous epithelium, and the premature desquamation of cells into the lumen of the seminiferous tubules. The limited data concerning the effects of multidrug immunosuppressive regimens on rat testes confirm our observations^21^. Freus et al. (2023) reported that immunosuppressive drugs in three-drug protocols, in combination with CsA + MMF + Pred and FK-506 + MMF + Pred, which are administered for 2 weeks before pregnancy and during 3 weeks of pregnancy to female rats, cause degenerative changes in the seminiferous tubules and interstitial tissue of male rat offspring after in utero exposure^21^. Notably, however, in this study, the duration of drug administration was relatively short compared with that in our studies. Unfortunately, no other studies have investigated the effects of multidrug immunosuppressive regimens on testicular morphology.

Numerous studies conducted on animal models using CNIs in monotherapy have confirmed their adverse effects on the male gonad. The authors noted that CsA administration causes changes in the hypothalamic‒pituitary‒gonadal axis and structural abnormalities in both the seminiferous tubules and the interstitial tissue^4,23–28^. The administration of CsA has been associated with histopathological abnormalities^4,23^ and reduction in sperm count following exposure to CsA^24^.

Other studies have likewise demonstrated that FK-506, when administered as monotherapy, has adverse effects on the male reproductive system^4,14^. Tacrolimus, like CsA, has been shown to affect the hypothalamic‒pituitary‒gonadal axis. Caneguim et al. (2009) reported that the administration of FK506 led to structural alterations in the seminiferous epithelium and peritubular compartment^14^. In rats treated with FK-506, other authors reported mild histological alterations within the seminiferous tubules, along with a modest reduction in both sperm counts^4^. Although FK-506 is 50–100 times more potent than CsA is, certain studies have reported that the associated testicular alterations are less pronounced than those induced by CsA^4^.

In our study, the conversion of treatment from CNIs to SRL monotherapy did not cause an increase in the changes in the seminiferous tubules in the rat testes. These changes were comparable to those observed in animals that received CNIs via multidrug protocols for 6 months without conversion. Unfortunately, very few studies have investigated the effects of the conversion of CNIs to SRL monotherapy on the male gonad^29^. He et al. (2013) reported that in kidney-transplanted rats subjected to conversion therapy from CsA to SRL, alterations in the lamina propria, disorganization of the seminiferous epithelium, and ultrastructural abnormalities of Sertoli cells were observed^29^. Available data on short-term administration of SRL in monotherapy have shown that this drug also adversely affects the hypothalamic‒pituitary‒gonadal axis and causes abnormalities in the seminiferous epithelium^4,15,16,30^.

Notably, in our study, the presented morphological changes were not severe and specific to the immunosuppressive drugs used. Similar changes have also been described in male gonads that were exposed to heavy metals^31^, inhibitors of aromatase P450^32^, or other factors disrupting spermatogenesis, e.g., diazacholesterol dihydrochloride^33^.

Structural alterations in the seminiferous tubules of male rat gonads resulting from the administration of immunosuppressive agents by multiple protocols may impair spermatogenesis and negatively affect semen quality, which consequently may lead to fertility disorders. The premature desquamation of germ cells into the tubular lumen may also indicate impaired Sertoli cell function.

Our findings indicate that immunosuppressive agents administered within multidrug regimens have adverse effects on testicular morphometric characteristics in rats. All the experimental groups exhibited a reduction in both the diameter and cross-sectional area of the seminiferous tubules. Moreover, a decrease in epithelial height was observed across all groups, apart from the TMP group.

Furthermore, a significantly lower percentage of Sertoli cells was observed only in the TMP group compared to the control group. There were no significant changes in the proportion of other cell types in experimental groups, indicating that germ cell differentiation was progressing normally. However, the number of Sertoli cells, spermatogonia, spermatocytes, and spermatids per tubule was lower in the CMP, CMP/S, and TMP/S groups. A decrease in the total cell count in this situation may suggest impaired spermatogenic efficiency and ultimately be associated with lower sperm count. The above-described morphological abnormalities likely contributed to the diminished morphometric values. To date, the effects of combined immunosuppressive therapies on testicular morphometry have not been thoroughly investigated in the available literature. Nevertheless, few data involving CNI monotherapy support the findings observed in our study. Caneguim et al. (2009) reported a marked decrease in both tubular and epithelial areas in the testes of rats administered FK-506^14^.

We also observed a lower diameter and area of seminiferous tubules in the FK-506-based group converted to SRL monotherapy vs. FK-506-based group without conversion. There were no significant differences in those parameters between the CMP/S and CMP groups. Our results agree with the findings of He et al.^29^. The authors revealed a reduction in the diameter of the seminiferous tubules after conversion from CsA to SRL. The remaining few reports on the effects of SRL monotherapy on the male gonad also confirmed the observed changes. In rats treated with SRL, the authors revealed a significantly reduced area, perimeter, and total and inner diameter of seminiferous tubules^15,16^.

In our study, in all the experimental groups, compared to the control group, greater accumulation of collagen around the blood vessels was detected (these changes were not statistically significant), whereas greater deposition of collagen fibres in the lamina propria was not detected. Notably, the thickness of the lamina propria (which is composed of a basement membrane, one layer of flattened myofibroblasts called peritubular contractile cells, fibroblasts and the extracellular matrix) of the seminiferous tubules remained unaltered, which explains the lack of severe alterations in the seminiferous epithelium. Scientific reports confirm that in the case of a thickened lamina propria, degeneration of the seminiferous epithelium should be expected^34^.

In the tunica albuginea (both the external part of this capsule, which is composed of dense irregular connective tissue, and the inner part, which is in the form of the tunica vasculosa, which is composed of a loose connective tissue layer containing blood vessels), visible changes were also not observed. In turn, increased collagen accumulation (statistically insignificant) in the perivascular space (in the absence of significant changes in the lamina propria) suggests the development of selective benign fibrosis.

Unfortunately, no studies have investigated fibrosis/collagen accumulation because of the use of immunosuppressive drugs in multidrug protocols. Only the few available data on the effects of CNIs used as monotherapies on the male gonad are available. The other authors confirmed our observations associated with an increase in collagen accumulation. The reported changes after the administration of CsA in interstitial tissue involve fibrosis and capillary congestion^4,25^.

Increased collagen accumulation around blood vessels within testicular interstitial tissue may impair vascular perfusion and limit the delivery of nutrients to selected cells. It can also be suggested that fibrotic remodelling may also be related to disruption of Leydig cell function, leading to reduced testosterone synthesis.

In our study, in animals treated with multidrug protocols based on CNIs, lower percentages of Ki67-positive cells and higher percentages of TUNEL-positive cells in the seminiferous tubules of male rat gonads were revealed than in the control group. Unfortunately, no studies have reported the percentage of cells with fragmentation of nuclear DNA and undergoing proliferation as a result of the administration of immunosuppressive drugs via triple protocols. The few available data concerning the effects of single drugs on the abovementioned parameters in the male gonad are limited.

The results of Caneguim et al. (2009) seem to confirm our observations. The authors also revealed TUNEL labelling in spermatogonia, spermatocytes, and round and elongate spermatids of the FK-506-treated rats^14^. However, the authors did not compare the percentage of TUNEL-positive cells between individual groups. In the case of proliferation, the findings from our previous research on the rat ventral and dorsal prostate glands^35,36^ appear to align with the current observations. Specifically, in the groups treated with CNI-based immunosuppressive regimens, we observed a higher percentage of TUNEL-positive cells, indicating increased apoptosis, both in the glandular epithelium and stroma of the rat prostate, than in the control animals^35,36^. These findings suggest that CNI-based therapies may simultaneously promote apoptotic process in the male reproductive system. Importantly, these proapoptotic effect might be more pronounced if CNIs are administered as monotherapies. The use of CNIs in multidrug combinations, as in the studied regimens, could mitigate these effects, potentially offering therapeutic advantages by balancing the rates of apoptosis and proliferation. This notion aligns with observations in other organs, such as the salivary glands, where long-term CNI administration via triple-drug protocols also induced morphological changes, including increased apoptosis, and collagen accumulation^13^. Moreover, CNIs have been associated with increased risks of certain cancers^37^, including prostate cancer, possibly because of their complex effects on cell death and proliferation pathways.

We also detected a lower percentage of Ki67-positive cells and TUNEL-positive cells in the seminiferous tubules of the male gonads of the rats, in which treatment was converted from CNIs to SRL. No studies have addressed the issue of the proliferation of seminiferous epithelial cells after conversion to SRL. However, few data on the effect of SRL in monotherapy seem to confirm our observations. Liu et al. (2017) reported that Ki67-positive cells within seminiferous tubules were markedly decreased in the testes of SRL-treated groups^16^. Moreover, the authors noted that the expression of Ki67 was associated with the degree of atrophy in the seminiferous tubules of the studied animals. In our earlier investigations on the rat prostate^35,36^, we reported that conversion from CNI-based protocols to SRL results in notable antiapoptotic and antiproliferative properties, which is consistent with the results of this report.

Ki67, a proliferation marker and downstream target of mTOR, is important in the context of identifying spermatogenesis disorders. In our study, we observed a reduction in the number of germ cells, which indicates spermatogenesis dysfunction. Notably, the decreased number of Ki-67-positive cells confirmed our observations.

Although the presented study has many limitations, the obtained results provide new and valuable data in this area. To fully explain both the changes themselves and their impact on functional reproductive parameters, additional semen and oxidative stress analysis and sex hormone measurements, particularly testosterone levels, are necessary in the future. The results presented in this study are promising and constitute an important starting point for further research in this area.

It is well known that the use of a rat model also presents inherent limitations, as results obtained in rodents cannot be directly extrapolated to humans. Nevertheless, in the context of investigating the effects of immunosuppressive drugs on gonadal function, studies conducted in animal models, such as rats, may hold particular significance. However, referring to studies conducted in animals, it is imperative to take into account the inherent metabolic and functional distinctions that characterize distinct species. It may be suggested that the findings of our study may offer a supplement to the formulation of strategies for fertility monitoring in men.

## Conclusions

The long-term administration of CNIs in multiple regimens influence the course of spermatogenesis in the testes of rats, which manifests as morphological alterations in the seminiferous epithelium, decreased total germ cells count, decreased proliferation of germinal cells and increased nuclear DNA fragmentation related to the apoptotic process. The conversion of treatment with CNIs to SRL also had adverse effects on the seminiferous epithelium; however, it had positive antiapoptotic effect. In consequence these changes may be associated with lower sperm count in semen.

Considering the morphological, morphometric and immunohistochemical findings, we can speculate that the observed changes in seminiferous tubules may result from the compromised adhesion junctions in the seminiferous epithelium. Cellular adhesive junctions play a fundamental role in preserving the structural integrity, shape, and volume of the seminiferous tubules. These specialized cell‒to-cell connections are primarily found between adjacent Sertoli cells, which form the most important element of the blood‒testis barrier, and between Sertoli and germ cells (apical specialization)^38^. One of the most notable consequences of decreased integrity of adhesion junctions is detachment of nonmature germ cells from the supporting Sertoli cells, which can lead to a reduction in the size and diameter of seminiferous tubules. The loss of these cells may also be associated with increased apoptosis or other forms of cell death and decreased proliferation.

### Clinical implications

Individuals after organ transplantation, who take immunosuppressive medications, and who are planning to become fathers are in a distinctive clinical situation that necessitates meticulous risk assessment and management by a team with extensive expertise in both transplantology and andrology. The use of medications such as calcineurin inhibitors and mTOR inhibitors has been associated with documented adverse effects on testicular structure and function, potentially resulting in impaired spermatogenesis. However, when administering MMF, additional caution is necessary. The European Medicines Agency (EMA) has issued recommendations stipulating that sexually active men undergoing treatment with MMF should employ contraceptive measures during the treatment period and for a 90-day follow-up period^39^. In practice, this entails meticulous planning and anticipation, or the consideration of alternative treatment regimens. It is important to acknowledge that the patient’s condition subsequent to organ transplantation and the presence of comorbidities can also exert an influence on male fertility.

Individuals who are undergoing organ transplantation should engage in informed planning for offspring and collaborate with specialists in transplantology, andrology, and endocrinology. Such efforts must encompass meticulous monitoring of testicular function, comprehensive hormonal testing, and seminal studies. However, it should be emphasized that therapeutic decisions must primarily focus on measures aimed at optimizing immunosuppressive therapy and preventing acute rejection of the transplanted organ.

## Methods

### Ethics statement

The experimental protocols were approved by the Local Commission of Ethics for the Care and Use of Laboratory Animals of Pomeranian Medical University in Szczecin, Poland (Resolution No. 24/08 of November 24, 2008 and Resolution No. 26/2011 of December 16, 2011). All animal experiments were performed in accordance with relevant guidelines and regulations, and in strict agreement with good animal practice with the recommendations in the Guide for Care and Use of Laboratory Animals and ARRIVE guidelines.

### Animals

The study was conducted on 30 sexually mature three-month-old male Wistar rats obtained from a licenced breeder (the Institute of Occupational Medicine, Lodz, Poland). The health and genetic certificates of the animals were approved by a veterinarian. Before the experiment, all the rats underwent a 2-week adaptation period. The animals were housed in standard cages (six rats per cage). The housing temperature was 21 °C ± 2 °C, the room humidity was maintained at 55 ± 5%, and the dark/light cycle was controlled by automatic timers and was set at 12 h light/12 h dark. The animals had free access to water and received a specialized laboratory diet linseed meal (LSM) type (Agropol Motycz, Lublin, Poland).

The rats were divided into five groups (*n* = 6 in each group): the control group and four experimental groups (CMP, CMP/S, TMP, and TMP/S). Animals in the control group received bread balls without any medication. The rats in the experimental groups were administered the following immunosuppressive drugs: CsA, FK-506, MMF, prednisone, and SRL, according to the three-drug protocols used in clinical practice after vascularized organ transplantation (Table 3).

**Table 3:** Drugs administered to the control and experimental groups.

Group	Drugs	Pharmaceutical form (name, manufacturer, city, country)	Dose (mg/kg body mass/day)	Time of administration (months)
C	–	–	–	–
CMP	CsA	Sandimmum-Neoral; Novartis International AG, Basel, Switzerland CellCept,	5.0	6
	MMF	Hoffman-La Roche Ltd., Basel, Switzerland	20.0	
	Prednisone	Encorton, Polfa, Pabianice, Poland	4.0	
CMP/S	CsA	Sandimmum-Neoral; Novartis International AG, Basel, Switzerland CellCept,	5.0	3
	MMF	Hoffman-La Roche Ltd., Basel, Switzerland	20.0	
	Prednisone	Encorton, Polfa, Pabianice, Poland	4.0	
	Sirolimus	Rapamune, Pfizer, Inc., New York, NY, USA	0.5	3
TMP	FK-506	Prograf, Astellas Pharma, Tokyo, Japan	4.0	6
	MMF	CellCept, Hoffman-La Roche Ltd., Basel, Switzerland	20.0	
	Prednisone	Encorton, Polfa, Pabianice, Poland	4.0	
TMP/S	FK-506	Prograf, Astellas Pharma, Tokyo, Japan	4.0	3
	MMF	CellCept, Hoffman-La Roche Ltd., Basel, Switzerland	20.0	
	Prednisone	Encorton, Polfa, Pabianice, Poland	4.0	
	Sirolimus	Rapamune, Pfizer, New York, NY, USA	0.5	3

C, control group without any medication; CMP, rats treated with cyclosporin A, mycophenolate mofetil, and prednisone for 6 months; CMP/S, rats treated with cyclosporin A, mycophenolate mofetil, and prednisone for the first 3 months of the experiment and sirolimus for the last 3 months; TMP, rats treated with tacrolimus, mycophenolate mofetil, and prednisone for 6 months; TMP/S, rats treated with tacrolimus, mycophenolate mofetil, and prednisone for the first 3 months of the experiment and sirolimus for the last 3 months.

For 6 months, the animals received oral immunosuppressive drugs in their pharmaceutical form in bread balls daily. The drug doses were adjusted to the body mass of the rats and were properly calculated while accounting for the metabolic differences between the rats and humans. The drug concentrations in the blood of the rats were within the therapeutic range as previously published by Grabowska et al.^22^.

### Euthanasia and collection of material

All the animals completed the study. The euthanasia of the experimental animals was performed in accordance with applicable ethical and legal standards under the direct supervision of a veterinarian. The rats were divided into two groups and underwent the procedure on two consecutive days to ensure optimal conditions. Ketamine hydrochloride (Ketalar, Pfizer, New York, NY, USA) was used as the anesthetic and was administered intraperitoneally at a dose of 50 mg/kg of body weight. Euthanasia was performed in a separate room dedicated to this purpose, which minimized stressful stimuli and eliminated the possibility of contact between the animals undergoing the procedure and other individuals.

During sectioning, the testes of the animals were obtained, routinely fixed in Bouin solution, and embedded in paraffin blocks. Next, 3-μm sections were cut for further analysis.

### Morphological studies

Sections of the testis were deparaffinized with xylene and rehydrated in a graded ethyl alcohol series (99.8–50%) (v/v). Next, the sections were stained using standard methods: haematoxylin and eosin (H&E) for routine histological examination, periodic acid Schiff (PAS) to visualize the lamina propria and different stages of the seminiferous tubule epithelium cycle, and Masson’s trichrome to reveal the collagen fibres.

### Immunostaining of Ki67 antigen–proliferation of cells

The immunostaining procedure was performed according to the protocol previously described in detail by Grabowska et al.^22^. The testis sections were boiled in Target Retrieval Solution (S2368; Dako, Glostrup, Denmark) at pH 9.0 for 30 min to reveal antigens. Endogenous peroxidase activity was blocked with peroxidase-blocking solution (S2023; Dako, Glostrup, Denmark). To determine the immunolocalization and immunoexpression of Ki67, a mouse monoclonal anti-Ki67 antigen primary antibody (IR626; clone MIB-1; Dako, Glostrup, Denmark; 1:100) was used. The specificity of immunostaining was confirmed by following the above procedures by replacing the primary antibody with IgG from mouse serum.

### TUNEL assay–nuclear DNA fragmentation

A terminal deoxynucleotidyl transferase dUTP nick-end labelling (TUNEL) assay was performed in accordance with the manufacturer’s guidelines (S7100; ApopTag Peroxidase In Situ Apoptosis Detection Kit; Millipore, Billerica, MA, USA) as previously described in detail by Grabowska et al.^35^. The negative controls for reaction specificity were performed by replacing the reaction mixture with terminal deoxynucleotidyl transferase (TdT; Millipore, Billerica, MA, USA) with label solution only. The sections were examined using a light microscope (Olympus BX 41, Hamburg, Germany).

### Quantitative analysis of morphological parameters, collagen and immunohistochemistry

H&E-, PAS-, and Masson trichrome-stained and TUNEL-, Ki67-immunostained slides were scanned at a magnification of 400 × on an Aperio AT2 digital slide scanner (Leica Microsystems, Wetzlar, Germany). The scanning resolution was 0.25 μm/pixel. The background illumination levels were calibrated using a prescan procedure. Moreover, the scanner was configured to minimize focus problems. The obtained digital slides of the tissues were examined on a computer screen using the ImageScope viewer software (version 11.2.0.780; Aperio Technologies, Vista, CA, USA). Other parameters were set to achieve compliance with the visual evaluation.

The diameter of the seminiferous tubules (μm) and the height of the seminiferous epithelium (μm) were assessed using a ruler tool, whereas the area of the seminiferous tubules was assessed using a pen tool. In each group, 90 seminiferous tubules were analysed (15 from each rat) in H&E- and PAS-stained tissue sections. Only cross-sections were analysed, and oblique and longitudinal sections were excluded.

The quantities of Sertoli cells, spermatogonia, spermatocytes, and spermatids were manually determined in cross-sections of seminiferous tubules. For each animal, counts were performed on 10 tubule sections (60 tubules per group), and the percentage of each cell type was calculated. Only Sertoli cells displaying characteristic nuclear morphology with a clearly visible nucleolus were included in the count. Given that germ cell numbers in rat vary between tubule stages I–VIII and IX–XIV, five tubules from stages I–VIII and five from stages IX–XIV were analyzed for each animal. The percentage of seminiferous tubule disorders (inappropriate arrangement of germ cells, the presence of prematurely sloughed cells into the lumen, markedly reduced number of germ cells, empty spaces in the epithelium, intercellular vacuolization, multinuclear cells, and irregular and folded walls) were also manually determined in 57–99 cross-sections of seminiferous tubules in each animal.

For the quantitative analysis of collagen fibres in rat testes stained with Masson’s trichrome, a positive pixel count v9 algorithm (version 9.1; Aperio Technologies, Vista, CA, USA) was used. The area of the blue-stained collagen fibres was measured and the percentage of collagen fibres was calculated. The areas of analysis were manually determined (cross-sections through the testis, including seminiferous tubules, interstitial tissue, and tunica albuginea). The percentage of collagen fibres was calculated in 6 slides in each group (cross-section through the entire testis with tunica albuginea).

For the quantitative analysis of TUNEL- and Ki67-positive cells in the testis, a nuclear v9 algorithm (version 9.1; Aperio Technologies, Vista, CA, USA) was used. The areas of analysis were also manually determined. The percentage of positive nuclei in the seminiferous epithelium was determined using the algorithm. The total number of positive nuclei was counted in 90 seminiferous tubules in each group (15 seminiferous tubules from each rat).

### Statistical analysis

All the statistical analyses were performed by using TIBCO Statistica version 13.3 (TIBCO Software, Palo Alto, CA, USA). The arithmetical means, standard deviations, medians and ranges were calculated. To assess data distribution, the Shapiro‒Wilk test was used. Data for testis weight, and percentage of tubules with markedly reduced number of germ cells, empty spaces in the epithelium, intercellular vacuolization, multinuclear cells and irregular and folded walls met the assumptions of normal distribution and equal variances (Brown-Forsyth test), therefore one-way analysis of variance and Tukey’s post hoc test were used. Since the other obtained values failed the normal distribution assumption, the nonparametric Kruskal‒Wallis test followed by Dunn’s multiple comparison test for post hoc analysis was used to assess the differences between the groups. A probability of *p* ≤ 0.05 was considered statistically significant.

## Data Availability

The data generated during this study are included in this article.  The dataset which supports the findings from this study is freely available at: https://ppm.pum.edu.pl/info/researchdata/PUM6fe3bd3c6ea0472197064ce4f65e9fc2/
